# Promoting Student Wellness and Self-Care During COVID 19: The Role of Institutional Wellness

**DOI:** 10.3389/fpsyt.2021.797355

**Published:** 2021-12-22

**Authors:** Marie Vazquez Morgan

**Affiliations:** Louisiana State University Health Shreveport, Shreveport, LA, United States

**Keywords:** COVID-19, medical students and residents, self-care, burnout, poor mental health

## Abstract

Stress and burnout are serious and growing threats to the mental health of medical trainees. Recent estimates of burnout in medical students and residents are quite high, with more than half displaying signs of stress, anxiety and depression. The COVID-19 pandemic has only heightened the state of poor mental health in these student populations. It is the position of LSU Health Shreveport Office of Institutional Wellness that a critical need exists for academic institutions to evaluate challenges to self-care and wellbeing in medical trainees. Such evaluations may pave the way for the development of effective institutional wellness initiatives and strategies, with the goal of reducing barriers to self-care to promote better mental and physical health, and facilitate improved quality of life in medical students and residents.

## Introduction

The COVID-19 pandemic created widespread stress and anxiety in the US population. Social distancing, increasing joblessness and limited access to mental health services, created an increased mental health burden during and beyond the pandemic (1–7). Student mental health has been a growing issue in academia. Even prior to the pandemic, national data sources displayed increased rates of burnout, depression, eating disorders, alcohol abuse and self harm in student populations (8). According to the 2019 Annual Report of the Center for Collegiate Mental Health, anxiety continued to be a widespread problem with numbers as high as 62.7% (9). The pandemic has only worsened students' mental health, with studies reporting increases in alcohol dependence, burnout, depression, suicidality, and fatigue (10–12). Studies have also displayed medical trainees may also be less likely to seek treatment due to the stigma of mental illness, and the perception of not being able to handle the rigors of medical training (10, 13).

Students of color (SOC) have been disproportionately impacted during the pandemic, displaying poorer outcomes with COVID-19 as well as increased rates of anxiety and depression (12, 14). Poorer mental health in SOC is often due to underlying social inequities, such as food and housing insecurity, racial and ethnic discrimination, and decreased access to technology required for online learning (12, 14).

One tactic to promote wellness and reduce stress during the rigors of medical training is to engage in self-care activities (15, 16). These activities should address the varied dimensions of wellness including physical activity, socialization, proper nutrition and stress management (17). However, the demanding pace of medical training often makes it a challenge for students to prioritize the time required for self-care activities (18).

Ayala et al., in a 2017 study on self-care, found “a robust inverse relationship between perceived stress and medical students' quality of life” (17). The authors concluded that their findings suggested self-care may be an effective strategy for reducing the effects of stress and burnout in medical students.

In addition, early application of self-care during medical training may promote sustainable healthy behaviors post-graduation (19). Sustainability of self-care is important post-graduation, as the literature reports that healthcare workers, including physicians, have experienced impaired mental health during the COVID-19 pandemic (20, 21). Protection of the mental health of physicians and other healthcare workers has been labeled as important by the World Health Organization in 2020 (22).

Due to the increased prevalence of mental health impairments in medical students and residents, investigating the relationships between poor mental health and the challenges faced in initiating self-care may assist academic institutions to better understand these issues and offer more effective wellness initiatives that address the barriers currently faced by trainees.

## Position

It is the position of LSU Health Shreveport, Office of Institutional Wellness that medical student and resident burnout is a significant issue, and lack of self-care is often overlooked as a contributing factor. A pressing need exists to evaluate the role of self-care to increase overall wellbeing and to combat burnout and stress during medical training. LSU Health Shreveport is committed to reducing student burnout by assessing and addressing the issues causing and fueling poor mental student health and by providing an academic environment that values wellness and self-care.

## Discussion

Medical trainees commonly experience stress and burnout, and poor mental health greatly contributes to decreases in their quality of life (9, 11, 13, 23). Researchers and wellness advocates maintain not practicing self-care during medical training is detrimental to mental and physical health and further contributes to increased risk of burnout and stress (15–17).

Promotion and facilitation of self-care during medical training is vital in order to promote long-term lifestyle modification that is sustainable post-graduation. This is important, as the literature displays that the mental health of medical professionals, not just medical students, has been challenged during the pandemic (20, 21, 23, 24). Further, poor mental health has been shown to be exacerbated when medical professionals feel unsupported by administration regarding wellbeing initiatives (20).

It is vital that academic institutions provide an organizational culture of wellbeing by assessing challenges to self-care and by implementing institutional strategies and measures that encourage sound mental and physical health. Such initiatives can include online wellness platforms, support groups, increased access to mental health services, classes on yoga, mindfulness, sleep health, time management, nutrition and fitness, as well as longitudinal wellness index surveys (25–27).

To facilitate self-care, virtual/in person classes on the importance of stress management, nutrition, sleep health and meditation can be incorporated into the matriculation process. Faculty and community wellness advocates can facilitate these classes during lunch hours or protected time allotted for self-care. Offering these types of classes during medical training is vital, as trainees whom increase their knowledge and practice self-care can more effectively weather school-related stress and build better resilience (28).

Longitudinal wellness index surveys can be used effectively to monitor stress and burnout (23, 25). These surveys provide complete anonymity to remove the fear of self-assessing, and trainees can compare their wellbeing measures to peers' and national averages. They can also gain access to local and national resources on stress/resiliency, work-life integration, and resources to combat or prevent alcohol/substance abuse to help improve their wellbeing. These surveys allow for continuous measurement through periodic reassessments in order for trainees to track their progress over time. They can also allow academic institutions to assess periods of high stress in trainees, to allow for better planning and scheduling of self-care activities.

In addition, addressing barriers to self-care is vital to ensure sound participation (18, 29). Due to the demanding pace of medical education and training, students and residents may perceive wellness activities as an encroachment on their time, which in turn may inadvertently increase stress levels and decrease their participation (18). Perceived loss of time from participating in wellness activities can be lessened through protected time for self-care activities. It can also be mitigated by incidental physical activity challenges. Incidental activity is defined as activity performed in small increments over the course of day. It tends to be less structured than a planned exercise session, and can occur in many forms (i.e. skipping the elevator and taking the stairs, walking during lunch or spending more time standing than sitting during the day). These activity challenges are easy to employ on a daily basis and students/residents may feel less threatened by them as they can be performed without further constraints on their time. Another way to address this barrier is to offer online wellness platforms that provide exercise and wellness classes on demand, so medical trainees can take classes when it best fits their schedules. Finally, sponsorships to local community gyms where trainees can meet to partake in team sporting tournaments and events can assist with increasing physical activity with the added benefit of socialization with peers.

Due to the COVID-19 pandemic, there has been an increased need for student mental health interventions, however due to time restrictive schedules, students and residents may not seek out treatment for poor mental health. To combat this challenge, behavioral health/counseling centers can add flexible hours (after hours or weekends), and provide a 24/7 crises management hotline to assist with emergent mental health issues.

Further, it is important to remember that the COVID-19 pandemic has not affected trainees equally, and has exacerbated the distinct mental health issues faced by SOC, lesbian, gay, bisexual, transgender, queer (LGBTQ) students, and students that are economically disadvantaged (12, 14). Academic institutional leadership needs to be aware of cultural issues that can impact both physical and mental wellbeing in underrepresented minority students and residents. Culturally based stigmas surrounding mental health care, an inherent distrust of the health care system or a lack of providers from diverse racial/ethnic backgrounds can be barriers to seeking out treatment. Providing counselors from diverse genders and backgrounds can assist with reducing these barriers and facilitate culturally competent care to all students. Further, staff should receive training on implicit bias, cultural awareness, sensitivity and competence. Cultural competence training is utilized to “increase therapists' awareness of their own assumptions, values, and biases and knowledge of research, assessment, and practice” (30). Such training can facilitate more equitable care and improve outcomes, particularly to students from culturally and linguistically diverse backgrounds (30, 31).

## Conclusions

Physical and emotional wellbeing are key to academic success in medical education. However, medical students and residents face poor mental health due to the rigors of their training and decreased ability to practice self-care. Research studies report medical trainees and healthcare professionals are at increased risk for poor nutrition, reduced sleep health, depression, and suicide. This is especially true in underrepresented students and residents. These conditions have become even more prevalent after COVID-19. Self-care has been shown to attenuate poor mental health and stress, however due to time constraints during medical training, few seek out or practice wellness activities. Increasing opportunities for medical trainees to participate in self-care activities during their training, can assist them in developing strong values for self-care, and become better wellness advocates to future patients. A fundamental shift recognizing the essential role of self-care is needed in order to address the epidemic of stress and burnout among medical trainees. Academic institutions must also understand and address the barriers students and residents face in regards to practicing self-care and wellbeing, and emphasize initiatives that address burnout-related issues and provide beneficial, culturally competent resources. Helping students and residents recognize the importance self-care and wellness during their medical training assists them in developing resilience, which will help them deal with stress more effectively during their matriculation and post-graduation, and improve their quality of life.

## Data Availability Statement

The original contributions presented in the study are included in the article/supplementary material, further inquiries can be directed to the corresponding author/s.

## Author Contributions

The author confirms being the sole contributor of this work and has approved it for publication.

## Conflict of Interest

The author declares that the research was conducted in the absence of any commercial or financial relationships that could be construed as a potential conflict of interest.

## Publisher's Note

All claims expressed in this article are solely those of the authors and do not necessarily represent those of their affiliated organizations, or those of the publisher, the editors and the reviewers. Any product that may be evaluated in this article, or claim that may be made by its manufacturer, is not guaranteed or endorsed by the publisher.
